# Expression of the angiogenic mediator, angiopoietin-like 4, in the eyes of patients with proliferative sickle retinopathy

**DOI:** 10.1371/journal.pone.0183320

**Published:** 2017-08-23

**Authors:** Kathleen Jee, Murilo Rodrigues, Fabiana Kashiwabuchi, Brooks P. Applewhite, Ian Han, Gerard Lutty, Morton F. Goldberg, Gregg L. Semenza, Silvia Montaner, Akrit Sodhi

**Affiliations:** 1 Wilmer Eye Institute, Johns Hopkins University School of Medicine, Baltimore, MD, United States of America; 2 Departments of Pediatrics, Medicine, Oncology, Radiation Oncology, Biological Chemistry, and Genetic Medicine, Johns Hopkins University School of Medicine, Baltimore, MD, United States of America; 3 Department of Oncology and Diagnostic Sciences, School of Dentistry; Department of Pathology, School of Medicine; Greenebaum Cancer Center, University of Maryland, Baltimore, MD, United States of America; Cedars-Sinai Medical Center, UNITED STATES

## Abstract

The recent success of therapies directly targeting the angiogenic mediator, vascular endothelial growth factor (VEGF), for the treatment of proliferative diabetic retinopathy has encouraged clinicians to extend the use of anti-VEGF therapies for the treatment of another ischemic retinal vascular disease, proliferative sickle cell retinopathy (PSR), the most common cause of irreversible blindness in patients with sickle cell disease. However, results from case reports evaluating anti-VEGF therapies for PSR have been mixed. This highlights the need to identify alternative therapeutic targets for the treatment of retinal neovascularization in sickle cell patients. In this regard, angiopoietin-like 4 (ANGPTL4) is a novel angiogenic factor regulated by the transcription factor, hypoxia-inducible factor 1, the master regulator of angiogenic mediators (including VEGF) in ischemic retinal disease. In an effort to identify alternative targets for the treatment of sickle cell retinopathy, we have explored the expression of ANGPTL4 in the eyes of patients with PSR. To this end, we examined expression and localization of ANGPTL4 by immunohistochemistry in autopsy eyes from patients with known PSR (n = 5 patients). Complementary studies were performed using enzyme-linked immunosorbent assays in aqueous (n = 8; 7 patients, 2 samples from one eye of same patient) and vitreous (n = 3 patients) samples from a second group of patients with active PSR. We detected expression of ANGPTL4 in neovascular tissue and in the ischemic inner retina in PSR, but not control, eyes. We further observed elevated expression of ANGPTL4 in the aqueous and vitreous of PSR patients compared to controls. These results suggest that ANGPTL4 could contribute to the development of retinal neovascularization in sickle cell patients and could therefore be a therapeutic target for the treatment of PSR.

## Introduction

Sickle cell disease is the most prevalent genetic hematologic disorder in the United States, disproportionately affecting African Americans[1]. Sickle cell patients who are homozygous for Hb S or who are heterozygous for Hb S and either Hb C or β-thalassemia are all at increased risk for vascular occlusions in the retina. These vaso-occlusions most often occur in the small vessels of the peripheral retina, can cause tissue ischemia[2, 3], and can lead to the development of retinal neovascularization (NV)[4]. These so-called neovascular “sea fan” lesions indicate presence of proliferative sickle retinopathy (PSR), the leading cause of vision loss in sickle cell patients[5, 6].

Scatter laser photocoagulation is the most commonly used intervention for PSR, with the long-term goal of preventing vision loss from a vitreous hemorrhage or a retinal detachment[7]. Scatter laser has been successfully used for the treatment of other ischemic retinopathies, most notably proliferative diabetic retinopathy (PDR)[8], retinopathy of prematurity (ROP)[9], and ischemic retinal vein occlusions (RVOs)[10]; nonetheless, clinical trials have not demonstrated a clear benefit for scatter laser in the treatment of retinal NV in sickle cell patients[11, 12]. Indeed, scatter laser leads to complete regression of only one-third of pre-existing retinal NV sea fans, a rate similar to their rate of spontaneous regression. Presently, reasonable disagreement exists as to whether (and when) treatment of NV in sickle cell patients with scatter laser results in better outcomes than observation alone[7], prompting exploration of other treatment options.

In this regard, we have recently demonstrated that expression of a hypoxia-regulated angiogenic factor, angiopoietin-like 4 (ANGPTL4)[13], is markedly increased in the eyes of patients with PDR[14, 15]. Like vascular endothelial growth factor (VEGF), we observed that ANGPTL4 expression is regulated by the transcription factor, hypoxia-inducible factor 1 (HIF-1), the master regulator of vasoactive factors in the ischemic retina [15]. We have further observed in pre-clinical studies that therapies targeting ANGPTL4 may augment therapies targeting vascular endothelial growth factor (VEGF) in treating retinal NV in the setting of PDR[14]. We hypothesize that ANGPTL4 may similarly contribute to the development of retinal NV in sickle cell patients. To interrogate this hypothesis, we have examined the expression of ANGPTL4 in the eyes of patients with PSR.

## Materials and methods

### Autopsy eyes

Institutional Review Board approval from the Johns Hopkins University School of Medicine was obtained for all autopsy eyes used in this study. 5 eyes from 5 sickle cell patients (documented by hemoglobin electrophoresis) with a history of untreated PSR (i.e., no history of scatter laser photocoagulation or prior intravitreal anti-VEGF therapy), and no known history of diabetes or ischemic retinal disease were selected for examination.

### Hematoxylin and Eosin (H&E) staining

After deparaffinization and hydration, tissue sections were stained with Harris hematoxylin (Polysciences Inc, Warrington, PA) for 20 seconds. The sections were then washed in distilled water, stained with lithium carbonate, rinsed in distilled water, and then stained in 0.5% alcoholic eosin (Polysciences Inc,). After dehydrating sections with xylene, cover slips were secured with Permount (Fisher Scientific, Waltham, MA).

### Immunohistochemistry

Immunohistochemical detection of ANGPTL4 (1:400 dilution; Abcam, Cambridge, MA) and CD34 (1:500 dilution; Covance, Princeton, NJ) was performed in paraffin-embedded tissue sections using premixed biotinylated anti-rabbit, anti-mouse and anti-goat immunoglobulins in phosphate buffered saline (PBS) from an ABC system (Dako, Santa Clara, CA) performed according to the manufacturer’s protocols as previously described[16, 17]. All immunohistochemical reagents, including antibodies, were identical for all specimens. Images were captured by scanning slides using the Aperio ScanScope program on Aperio Scanscope XT® System (Leica Biosystems, Wetzlar, Germany).

### Aqueous and vitreous samples

Institutional Review Board approval from the Johns Hopkins University School of Medicine (Baltimore, MD) was obtained for all patient samples used in this study (NA_00084367, NA_00073289, and NA_00071340). Inclusion criteria for PSR and PDR patients included a known diagnosis of sickle cell disease (by hemoglobin electrophoresis) or diabetes, respectively, and the presence of active retinal neovascularization (observed on clinical exam or fluorescein angiography). Exclusion criteria for control patients included diabetes, sickle cell disease, or any ischemic retinal disease. Exclusion criteria for all patients included any other ischemic retinal disease, uveitis, retinal detachment within 1 year of sample collection, or neovascularization from another cause. Ocular samples were collected from consenting patients at the Wilmer Eye Institute undergoing intravitreal injection for active neovascularization (aqueous samples for PSR or PDR eyes), cataract surgery (aqueous samples for control eyes), or vitrectomy surgery for non-clearing vitreous hemorrhage (vitreous biopsies). Aqueous samples were obtained immediately after performing intravitreal injection or immediately prior to initiating cataract surgery (using a 30 gauge needle). Undiluted vitreous biopsies were obtained at the beginning of vitrectomy surgery. Consent was written and voluntary without stipend. Aqueous samples were immediately processed and stored at -80°C prior to analysis. Vitreous samples were immediately centrifuged at 16,000 x g for 5 min at 4°C, and then the supernatant was stored at -80°C prior to analysis.

### ELISA

ANGPTL4 (DuoSet) and VEGF (Quantikine) ELISA kits were purchased from R&D Systems (Minneapolis, MN). Aqueous and vitreous were analyzed for ANGPTL4 (10 μL of aqueous diluted 1:10, 100 μL of undiluted vitreous) and VEGF (10 μL of aqueous or vitreous diluted 1:10) with ELISAs, performed according to the manufacturer’s protocols as previously described [14]. All ELISAs were performed in duplicate and quantitation was performed using the standard curve included in the kit.

### Statistical analysis

We estimated that the minimum biologically- (and clinically-) relevant increase of ANGPTL4 in PSR patients, compared to control patients (without ischemic retinal disease or NV from another cause), would be a 2-fold increase. For power calculations, our pilot studies demonstrated that aqueous and vitreous concentrations of ANGPTL4 are approximately 2 ng/mL in non-diabetic control patients. To detect a biologically relevant increase in the levels of ANGPTL4 in patients with PSR, we assigned μ1 = [ANGPTL4] in PSR patients = 4 ng/mL; μ2 = [ANGPTL4] in control patients = 2 ng/mL; and the common standard deviation for [ANGPTL4] in control patients, Σ = 0.8. Importantly, the latter takes into account the impact of the typical biological variability observed in biological samples (i.e., Σ = standard deviation). For α = 0.05 and power = 0.80, we calculated that we would need a minimum of 3 PSR patients to sufficiently power our study to observe a statistically significant, biologically-relevant increase in ANGPTL4 in PSR eyes.

Results from clinical samples are shown as mean ± SD. Statistical differences between groups were determined by one-way ANOVA and Holm-Sidak’s multiple comparisons tests. Correlations were tested using the Pearson method. Statistical analyses were performed using Prism 6.0 software (GraphPad).

## Results

### ANGPTL4 is expressed in retinal NV tissue in PSR eyes

**Fig 1:**
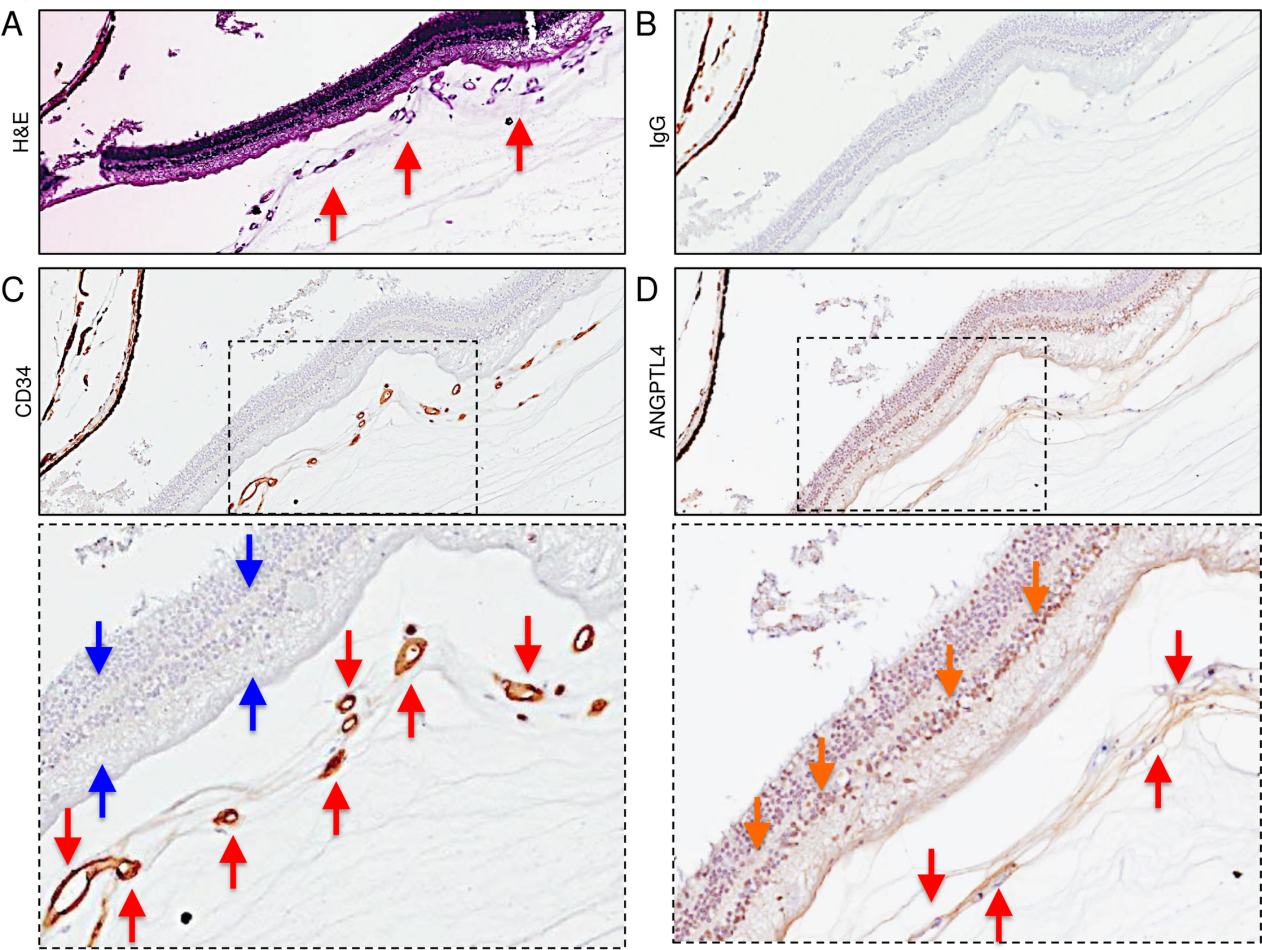
Expression of ANGPTL4 in the peripheral ischemic inner retina and within adjacent retinal neovascular tissue of PSR eyes. (**A**) H&E staining of proliferative vessels (red arrows) observed on the surface of the peripheral retina of a PSR eye. (**B**) Non-staining IgG (negative control). (**C**) CD34 staining (vascular endothelial cells) of proliferative vessels (red arrows) overlying non-perfused (i.e., no CD34 staining of retinal vessels) peripheral retina (blue arrows). (**D**) ANGPTL4 staining within non-perfused (i.e., no CD34 staining of retinal vessels) peripheral retina (orange arrows) and in the vascular endothelial cells and within the stroma (red arrows) of the retinal neovascular tissue overlying the peripheral non-perfused retina. Similar results were obtained in 5/5 PSR eyes tested.

To evaluate the expression of the HIF-regulated angiogenic mediator, ANGPTL4, in the eyes of patients with known PSR, we examined 5 paraffin-embedded autopsy eyes from 5 non-diabetic patients sickle cell disease with untreated PSR (i.e., no prior history of scatter laser photocoagulation or intravitreal anti-VEGF therapy), and no history of another ischemic retinal disease. We focused on areas of the peripheral retina anterior to the margin between perfused and non-perfused retina, as we have recently reported that VEGF and the α-subunit of HIF-1, the transcription factor that regulates its expression, are expressed in the peripheral ischemic retina in this region in PSR eyes[17]. We have also previously demonstrated that ANGPTL4 expression is regulated by HIF-1 in diabetic eye disease[13–15] as well as in the intraocular tumor, uveal melanoma[18], similar to VEGF. We observed strong expression of ANGPTL4 in the vascular endothelial cells lining neovascular sea fans (highlighted by expression of the endothelial cell marker, CD34) as well as in the stroma of the fibrovascular tissue in this region in 5/5 PSR eyes examined (Fig 1 and S1 Fig). We further observed expression of ANGPTL4 within the ischemic inner and outer retinal cells underlying the neovascular sea fans (Fig 1 and S2 Fig).

### ANGPTL4 is expressed in the peripheral ischemic retina distant from retinal NV in PSR eyes

**Fig 2:**
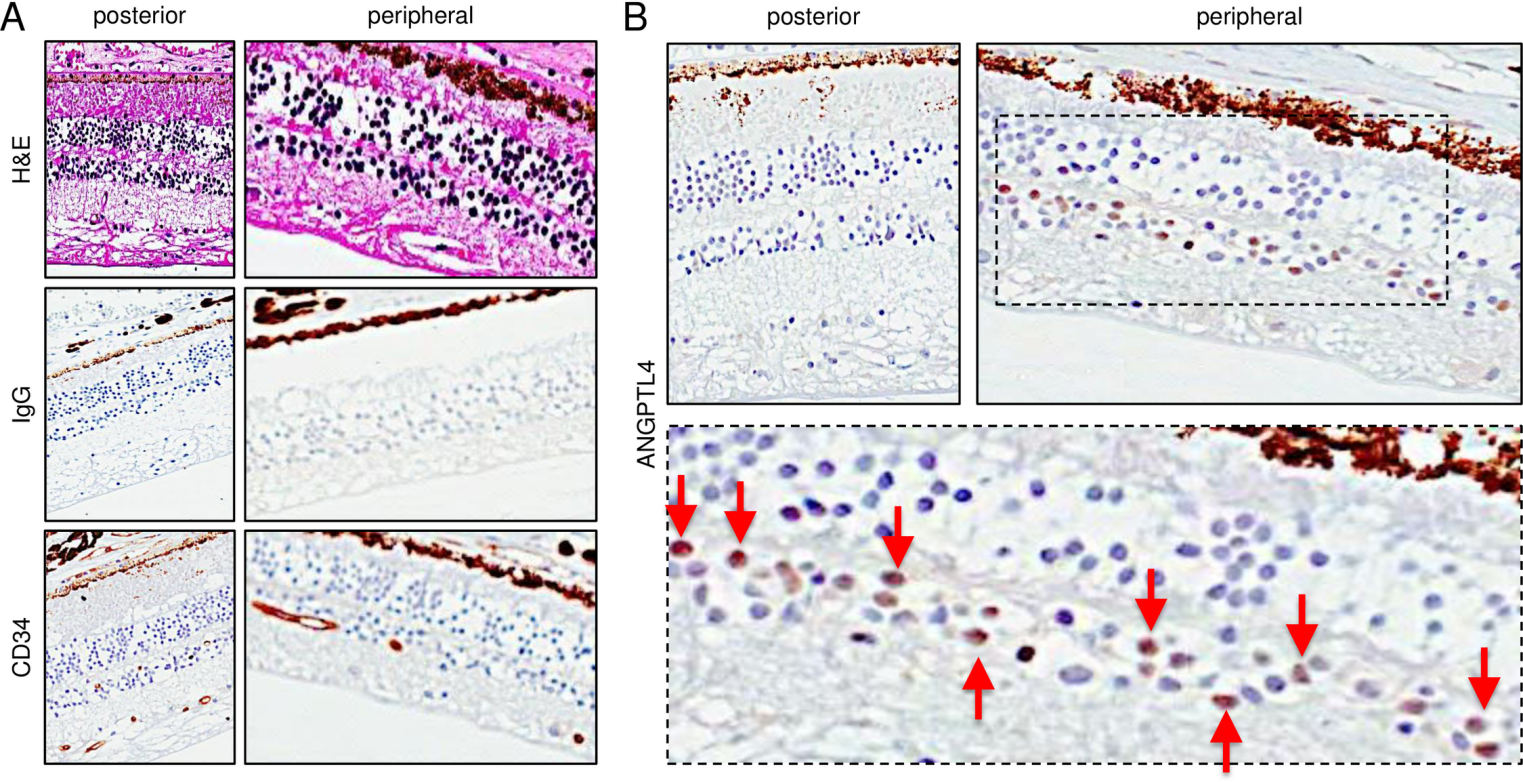
Expression of ANGPTL4 in the peripheral ischemic retina distant from retinal neovascular tissue of sickle cell retinopathy eyes. (**A**) H&E staining (*top*), IgG non-staining (*middle*; negative control), and CD34 staining (*bottom*) in the central (posterior) vs. peripheral (anterior) retina of a PSR eye in a region in which no adjacent retinal neovascularization is detected under low power magnification. Sparse CD34-positive inner retinal vessels, but no overlying neovascular tissue, are observed in the peripheral ischemic retina. (**B**) ANGPTL4 staining in the posterior vs. peripheral inner retina. Staining of ANGPTL4 is noted in the peripheral ischemic retina (red arrows) but not in the perfused posterior retina. Similar results were obtained in 5/5 PSR eyes tested.

We have previously reported that HIF-1α and VEGF are also expressed in the peripheral ischemic retina in regions without active NV in PSR eyes[17]. We therefore examined expression of ANGPTL4 in the ischemic peripheral retina of autopsy eyes from patients with sickle cell retinopathy in regions without active NV. We observed an absence of vascular structures, highlighted by the absence of expression of the endothelial cell-specific antigen, CD34, in the peripheral retina of 5/5 eyes with PSR. Within these regions of ischemic retina, we detected strong expression of ANGPTL4 in the inner retina (Fig 2). Importantly, we did not detect any expression of ANGPTL4 in the inner retina in the posterior, perfused regions of these same eyes (Fig 2).

### ANGPTL4 expression is increased in the aqueous of patients with active PSR

**Fig 3:**
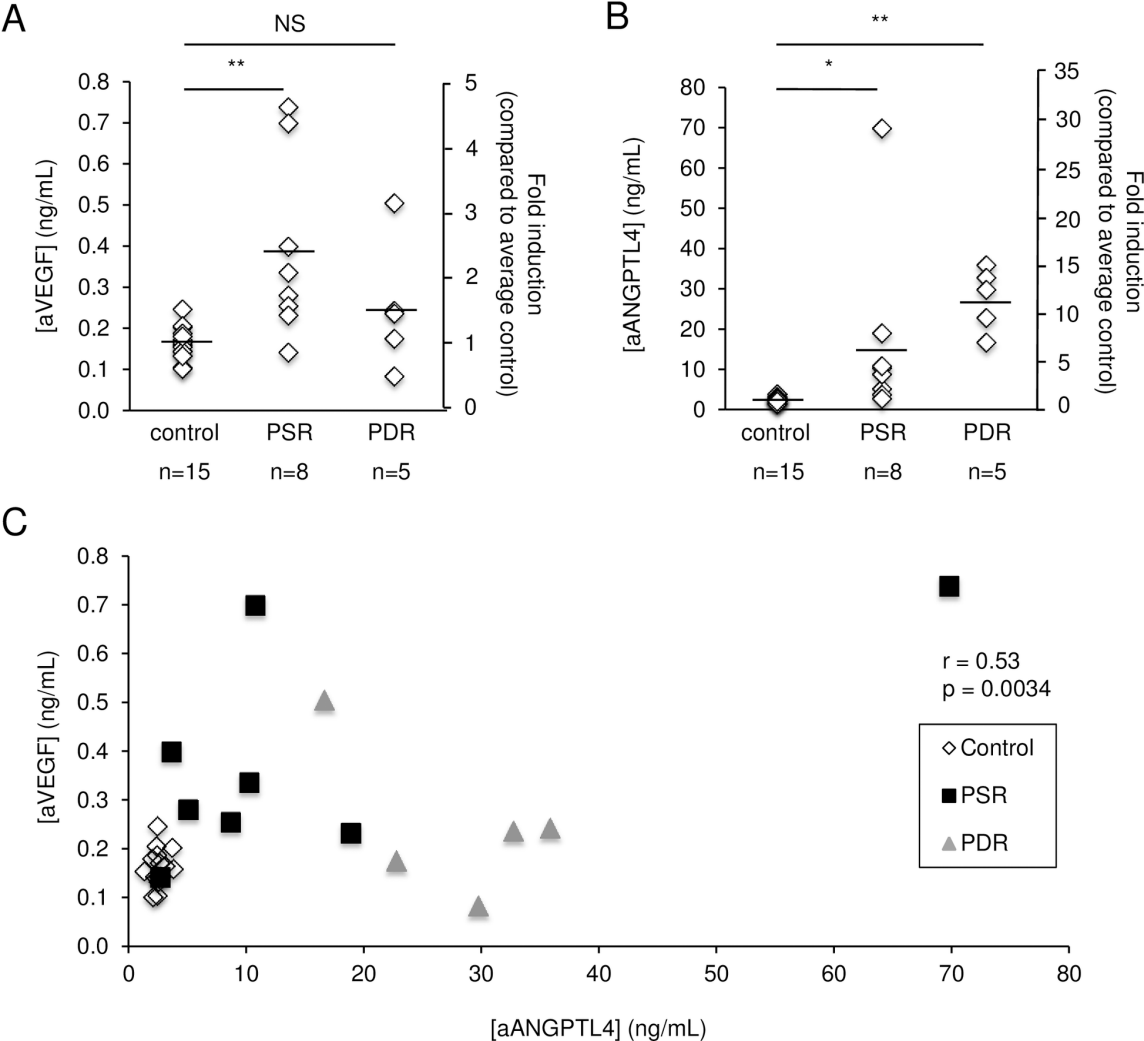
VEGF and ANGPTL4 expression in the aqueous of patients with active PSR. (**A** and **B**) Aqueous levels of VEGF (aVEGF) (**A**) and ANGPTL4 (aANGPTL4) (**B**) were measured in patients with active PSR and compared to control patients, as well as patients with active PDR. (**C**) Correlation between aVEGF and aANGPTL4 in control, PSR, and PDR patients. Control: non-diabetic, non-sickle cell patients; PDR: patients with active PDR. Aqueous levels of ANGPTL4 and VEGF are reported as concentration (ng/mL) or fold induction (compared to the average level for non-diabetic, non-sickle cell control patients, arbitrarily assigned as 1). NS = not significant; * p < 0.05; ** p < 0.01.

To determine whether secreted levels of ANGPTL4 are increased in the eyes of patients with PSR, we examined aqueous samples from 7 patients (8 samples total; 2 samples were obtained from the same patient at two different times during separate surgeries) with PSR (Table 1) for levels of ANGPTL4. The variability of the aqueous levels of both ANGPTL4 and VEGF was similar and represents the typical variability observed in biological samples. VEGF expression in the aqueous of PSR eyes (0.38±0.22 ng/mL; mean±SD) was increased 2.4-fold compared to control eyes (0.16±0.04 ng/mL) (Fig 3A). We similarly observed a marked increase in the expression of ANGPTL4 in the aqueous of PSR eyes (16.24±22.25 ng/mL) compared to control eyes (2.57±0.62 ng/mL) (6.3-fold; Fig 3B). ANGPTL4 levels were elevated in 8/8 aqueous samples from PSR patients, compared to the average ANGPTL4 level in control patients. These results were comparable to the increased expression of ANGPTL4 observed in the aqueous of patients with PDR (10.7-fold) (Fig 3B).

**Table 1:** Patient aqueous samples.

Patient	Age (years)	Sex	Phakic Status*	Prior Vitrectomy	Prior Laser	Genotype	DM Type	DM Duration (years)	CVD
**Control**									
1	72	F	P	No	No	–	–	–	No
2	75	F	P	No	No	–	–	–	Yes
3	68	F	P	No	No	–	–	–	Yes
4	49	F	P	No	No	–	–	–	Yes
5	76	F	P	No	No	–	–	–	Yes
6	83	M	P	No	No	–	–	–	Yes
7	71	F	P	No	No	–	–	–	Yes
8	61	M	P	No	No	–	–	–	No
9	73	M	P	No	No	–	–	–	No
10	60	F	P	No	No	–	–	–	No
11	54	F	P	No	No	–	–	–	No
12	75	F	P	No	No	–	–	–	Yes
13	57	F	P	No	No	–	–	–	No
14	64	F	P	No	No	–	–	–	No
15	85	F	P	No	No	–	–	–	Yes
**PSR**									
1	39	M	P	No	No	Unknown	–	–	No
2	58	M	P	No	Yes	SS	–	–	Yes
3§	54	M	P	Yes	Yes	SC	–	–	Yes
4§	54	M	P	Yes	Yes	SC	–	–	Yes
5	80	F	PP	Yes	Yes	AS	II	20	Yes
6	32	M	P	No	Yes	SC	–	–	No
7	52	F	P	No	Yes	SC	–	–	No
8	37	F	P	No	Yes	SS	–	–	No
**PDR**									
1	50	M	P	No	No	–	I	15	Yes
2	52	M	PP	No	No	–	II	Unknown	Yes
3	56	F	P	No	Yes	–	II	15	Yes
4	57	M	P	No	Yes	–	II	30	Yes
5	52	M	P	No	Yes	–	II	12	No

* At time of sample collection.

P, phakic. PP, pseudophakic. DM, diabetes mellitus. CVD, cardiovascular disease. PSR, proliferative sickle retinopathy. PDR, proliferative diabetic retinopathy. SS, homozygous for the hemoglobin S mutation. SC, heterozygous for the hemoglobin S and C mutation. AS, sickle cell trait.

§Sample from one eye of same patient.

There was a trend toward a positive correlation between the aqueous levels of ANGPTL4 and the aqueous levels of VEGF in PSR patients (r = 0.65, p = 0.078) (Fig 3C); however, this correlation was not statistically significant. There was no correlation between ANGPTL4 and VEGF aqueous levels in patients with PDR (r = -0.60, p = 0.29) or in non-diabetic control patients (r = 0.19, p = 0.49; Fig 3C).

### ANGPTL4 expression is increased in the vitreous of patients with active PSR

**Fig 4:**
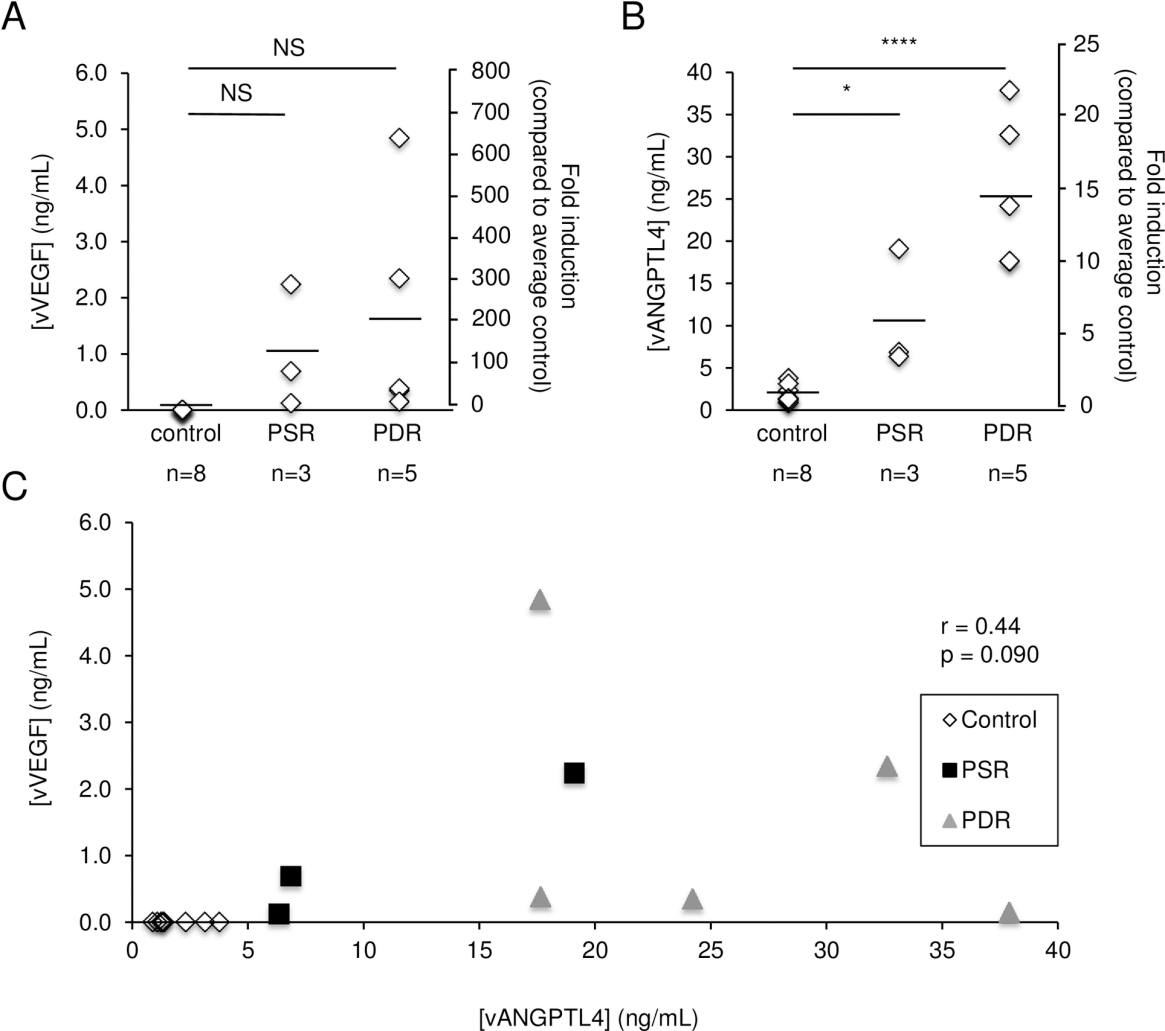
VEGF and ANGPTL4 expression in the vitreous of patients with active PSR. (**A** and **B**) Vitreous levels of VEGF (vVEGF) (**A**) and ANGPTL4 (vANGPTL4) (**B**) were measured in patients with active PSR and compared to control patients, as well as patients with active PDR. (**C**) Correlation between vVEGF and vANGPTL4 in control, PSR, and PDR patients. Control: non-diabetic, non-sickle cell patients; PDR: patients with active PDR. Vitreous levels of ANGPTL4 and VEGF are reported as concentration (ng/mL) or fold induction (compared to the average level for non-diabetic, non-sickle cell control patients, arbitrarily assigned as 1). NS = not significant; * p < 0.05; **** p < 0.0001.

Motivated by these promising results, we performed vitreous biopsies on sickle cell patients with active NV undergoing vitrectomy surgery (Table 2), and examined the expression of ANGPTL4 compared to control patients. Again, the variability of the vitreous levels of ANGPTL4 and VEGF were similar and represents the typical variability observed in biological samples. The average for the vitreous VEGF levels in PSR (1.02±1.10 ng/mL) was increased compared to control patients (0.02±0.03 ng/mL), as has been previously published[19] (Fig 4A). However, this difference was not statistically significant. We did observe elevated levels of ANGPTL4 in the vitreous of 3/3 PSR patients (10.76±7.23 ng/mL) compared to non-diabetic control patients (1.88±1.06 ng/mL) (Fig 4B). Importantly, this increase was statistically significant (p<0.05). The levels of ANGPTL4 in the vitreous of PSR patients were between 3.4- and 10.1-fold higher than the average level in the vitreous of non-diabetic controls. Although the number of patients was small, there was a trend toward a positive correlation between the vitreous levels of ANGPTL4 and the vitreous levels of VEGF in PSR patients (r = 0.97, p = 0.14) (Fig 4C). Similar to the aqueous levels, there was no correlation between ANGPTL4 and VEGF vitreous levels in patients with PDR (r = -0.37, p = 0.54) or in non-diabetic control patients (Fig 4C).

**Table 2:** Patient vitreous samples.

Patient	Age (years)	Sex	Phakic Status*	Prior Vitrectomy	Prior Laser	Genotype	DM Type	DM Duration (years)	CVD
**Control**									
1	70	M	P	No	No	–	–	–	Yes
2	62	F	PP	Yes	No	–	–	–	No
3	70	M	PP	No	No	–	–	–	Yes
4	47	F	P	No	No	–	–	–	No
5	86	F	PP	No	No	–	–	–	Yes
6	72	M	PP	No	No	–	–	–	Yes
7	55	M	P	No	No	–	–	–	No
8	46	M	PP	No	No	–	–	–	Yes
**PSR**									
1	54	M	P	Yes	Yes	SC	–	–	Yes
2	52	F	P	No	Yes	SC	–	–	No
3	37	F	P	No	Yes	SS	–	–	No
**PDR**									
1	50	M	P	No	No	–	I	15	Yes
2	52	M	PP	No	No	–	II	Unknown	Yes
3	56	F	P	No	Yes	–	II	15	Yes
4	57	M	P	No	Yes	–	II	30	Yes
5	52	M	P	No	Yes	–	II	12	No

* At time of sample collection.

P, phakic. PP, pseudophakic. DM, diabetes mellitus. CVD, cardiovascular disease. PSR, proliferative sickle retinopathy. PDR, proliferative diabetic retinopathy. SC, heterozygous for the hemoglobin S and C mutation. SS, homozygous for the hemoglobin S mutation.

## Discussion

The rationale for the use of scatter laser photocoagulation in PSR patients is supported by the successful use of this approach for treatment of neovascularization in PDR[8], ROP[9], and ischemic RVOs[10]. However, clinical trials for PSR patients have not demonstrated better outcomes for scatter laser photocoagulation than observation alone[7], encouraging clinicians to explore other treatment options. An emerging, alternative approach to scatter laser photocoagulation for the treatment of retinal NV is the direct inhibition of the secreted mediators that promote pathological angiogenesis. In a recent multi-center randomized clinical trial, for example, it was demonstrated that anti-VEGF therapy may be as effective as PRP for the treatment of patients with PDR[20]. We have previously reported that HIF-1α and VEGF are expressed in ischemic retinal tissue and overlying neovascular sea fans in PSR eyes, similar to PDR[17]. These results support the use of therapies targeting VEGF to treat retinal neovascularization in PSR patients.

The use of anti-VEGF therapy for PSR has been reported in only a handful of case reports, the results of which have varied[21–24]. Moreover, frequent hospitalizations for sickle cell crises, combined with development of PSR at a young age, may make PSR patients poor candidates for a therapy that may require long-term treatment with frequent (e.g., monthly) intravitreal injections. Whether short-term treatment with anti-VEGF therapy promotes auto-infarction of NV in a subset of PSR patients who would otherwise progress without treatment, remains unexamined.

One explanation for the infrequent efficacy of anti-VEGF therapy in PSR patients is the potential contribution of other angiogenic mediators to the development of retinal NV in these patients. In this regard, post hoc analyses of studies examining the role of anti-VEGF therapies in diabetic eye disease suggest that monthly anti-VEGF treatment may not be adequate to prevent the progression of diabetic retinopathy in all treated patients[25, 26]; this is consistent with a role for other angiogenic mediators in the pathogenesis and progression of diabetic eye disease. In this regard, in a preclinical study, we recently provided evidence supporting a role for neutralizing antibodies targeting ANGPTL4 in the treatment of PDR[14, 15]. Collectively, these observations suggest that therapies targeting secreted factors (in addition to VEGF) may be more effective than targeting only VEGF to treat retinal NV in patients with ischemic retinopathies.

Here we have demonstrated that ANGPTL4 is expressed in retinal neovascular sea fans and in the underlying ischemic inner retina in eyes with active PSR. We further report that the inner retina, in regions not immediately adjacent to neovascular sea fans, also demonstrates expression of ANGPTL4. Although increased expression of ANGPTL4 does not necessarily mean that this angiogenic mediator plays a pathologic role in PSR, our results provide a foundation for future studies examining the contribution of ANGPTL4 to the development of retinal NV in PSR patients. We speculate that expression of ANGPTL4 and VEGF at sites distal from sea fans may be sufficient to promote their survival following localized (sectoral) scatter laser. If this is correct, then more broad application of scatter laser photocoagulation, including circumferential scatter laser, would be required to quench the expression of these angiogenic mediators and treat patients with PSR[17].

**Fig 5:**
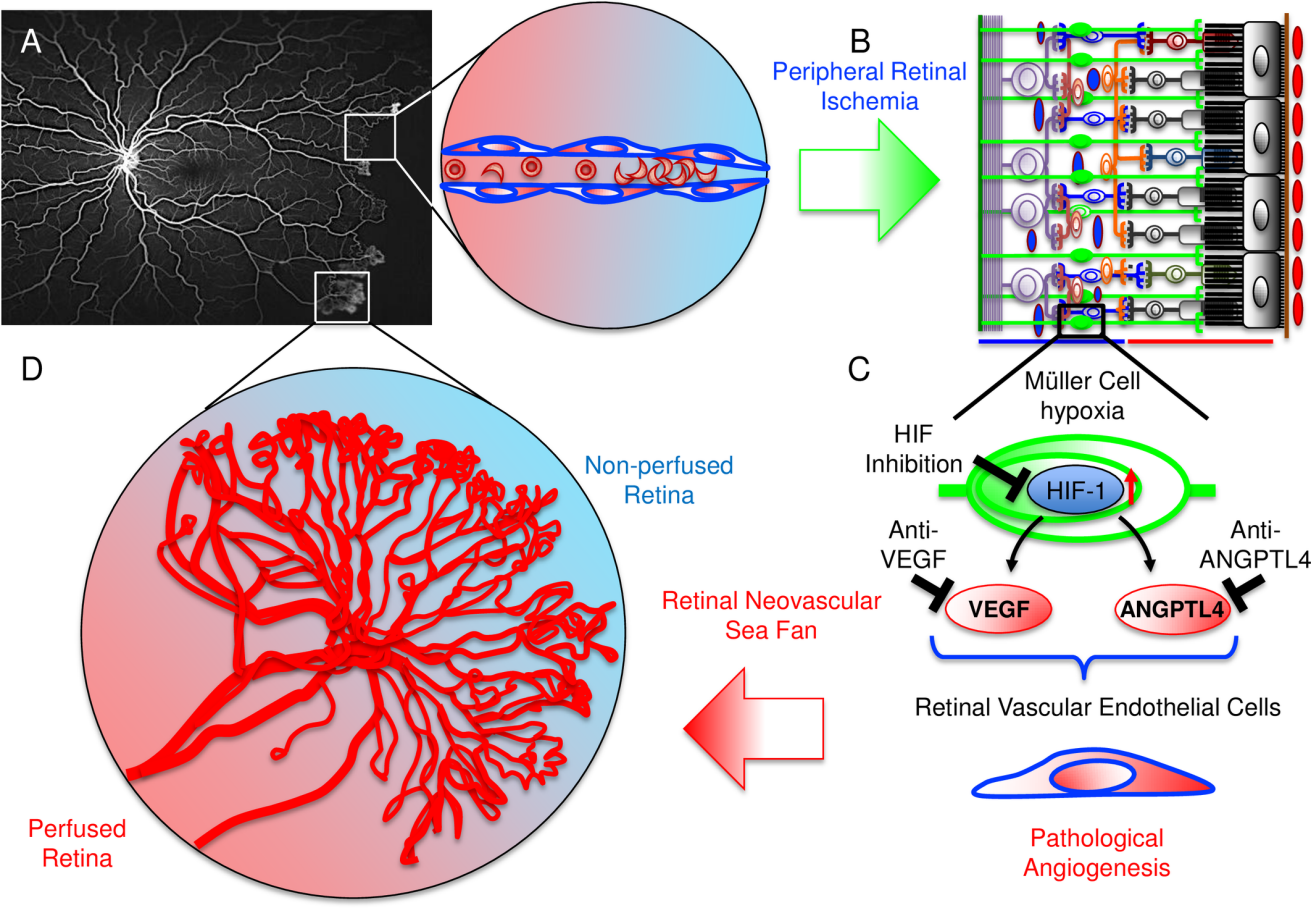
Schematic illustration depicting the progression from peripheral non-perfusion to the development of neovascular sea fans in PSR. (**A**) FA image from a patient with PSR demonstrating extensive peripheral non-perfusion and developing neovascular sea fans. Expanded view of local area of peripheral non-perfusion following occlusion of vessels by sickled red blood cells. (**B**) Schematic demonstrating inner retinal non-perfusion leading to Müller cell hypoxia. (**C**) Nuclear accumulation of HIF-1 leads to increased expression of both VEGF and ANGPTL4, which together act on retinal vascular endothelial cells to promote pathological angiogenesis. (**D**) This, in turn, results in the formation of neovascular sea fans at the margin between perfused and non-perfused retina. Inhibition of HIF-1 or both VEGF and ANGPTL4 could be an effective approach for the treatment of neovascular sea fans in sickle cell patients.

Although not statistically significant, the trend toward a positive correlation between aqueous and vitreous levels of VEGF and ANGPTL4 in PSR eyes suggests that increased expression of one may predict increased expression of the other. We speculate that in eyes in which both angiogenic mediators are elevated and contribute to the survival of retinal NV, therapies targeting only one of these factors may not be sufficient to adequately promote the regression of neovascular sea fans in PSR patients. An alternative approach would be to simultaneously target ANGPTL4 and VEGF, either by combining current anti-VEGF therapies with those that directly inhibit ANGPTL4 or by directly inhibiting the transcription factor, HIF-1, thereby blocking expression of VEGF and ANGPTL4, as well as additional angiogenic factors regulated by HIF-1 (Fig 5).

Strengths of this study include the qualitative assessment of ANGPTL4 expression in autopsy eyes from patients with PSR, combined with the quantitative assessment of ANGPTL4 in aqueous and vitreous samples from a separate group of sickle cell patients with active PSR (compared to non-sickle cell, non-diabetic control patients). Limitations of this study include the study size. To calculate the sample size for our studies, we assumed the minimum biologically-(and clinically-) relevant increase of ANGPTL4 in the aqueous or vitreous of PSR patients as compared to control patients, would be 2-fold. The number of samples included in our study met or exceeded the sample size that we calculated would be necessary to detect a statistically significant, biologically-relevant increase in ANGPTL4 in PSR patients, as compared to control patients. Indeed, we did detect a statistically significant increase in ANGPTL4 in both the aqueous and vitreous of PSR patients. Interestingly, we did not detect a similar statistically significant increase in VEGF in the vitreous of PSR (or PDR) patients; this is likely a consequence of the higher variability of the aqueous and vitreous concentrations of VEGF compared to ANGPTL4. In light of the poor response of PSR to current treatment options (including the limited case reports assessing the effects of anti-VEGF therapy in PSR patients), this study provides the groundwork for future studies to assess the relative contribution of ANGPTL4 to the development of pathological angiogenesis in PSR eyes.

## Supporting information

S1 Fig High magnification images demonstrating expression of ANGPTL4 in the peripheral ischemic inner retina and within adjacent retinal neovascular tissue of PSR eyes. **(A)** H&E staining of proliferative vessels (red arrows) observed on the surface of the peripheral retina of a PSR eye. **(B)** Non-staining IgG (negative control). **(C)** CD34 staining (vascular endothelial cells) of proliferative vessels (red arrows) overlying non-perfused (i.e., no CD34 staining of retinal vessels) peripheral retina (blue arrows). **(D)** ANGPTL4 staining within non-perfused (i.e., no CD34 staining of retinal vessels) peripheral retina (orange arrows) and in the vascular endothelial cells and within the stroma (red arrows) of the retinal neovascular tissue overlying the peripheral non-perfused retina.
(TIF)

S2 Fig High magnification images demonstrating expression of ANGPTL4 in the inner retina and within adjacent retinal neovascular tissue of PSR eyes. ANGPTL4 staining within inner retinal cells as well as in the vascular endothelial cells and within the stroma of the retinal neovascular tissue.
(TIF)
