# Supplementary figures and images for: Expression of the angiogenic mediator, angiopoietin-like 4, in the eyes of patients with proliferative sickle retinopathy
Source: PLoS One. 2017 Aug 23;12(8):e0183320. doi: 10.1371/journal.pone.0183320 (PMC5568377; doi:10.1371/journal.pone.0183320)

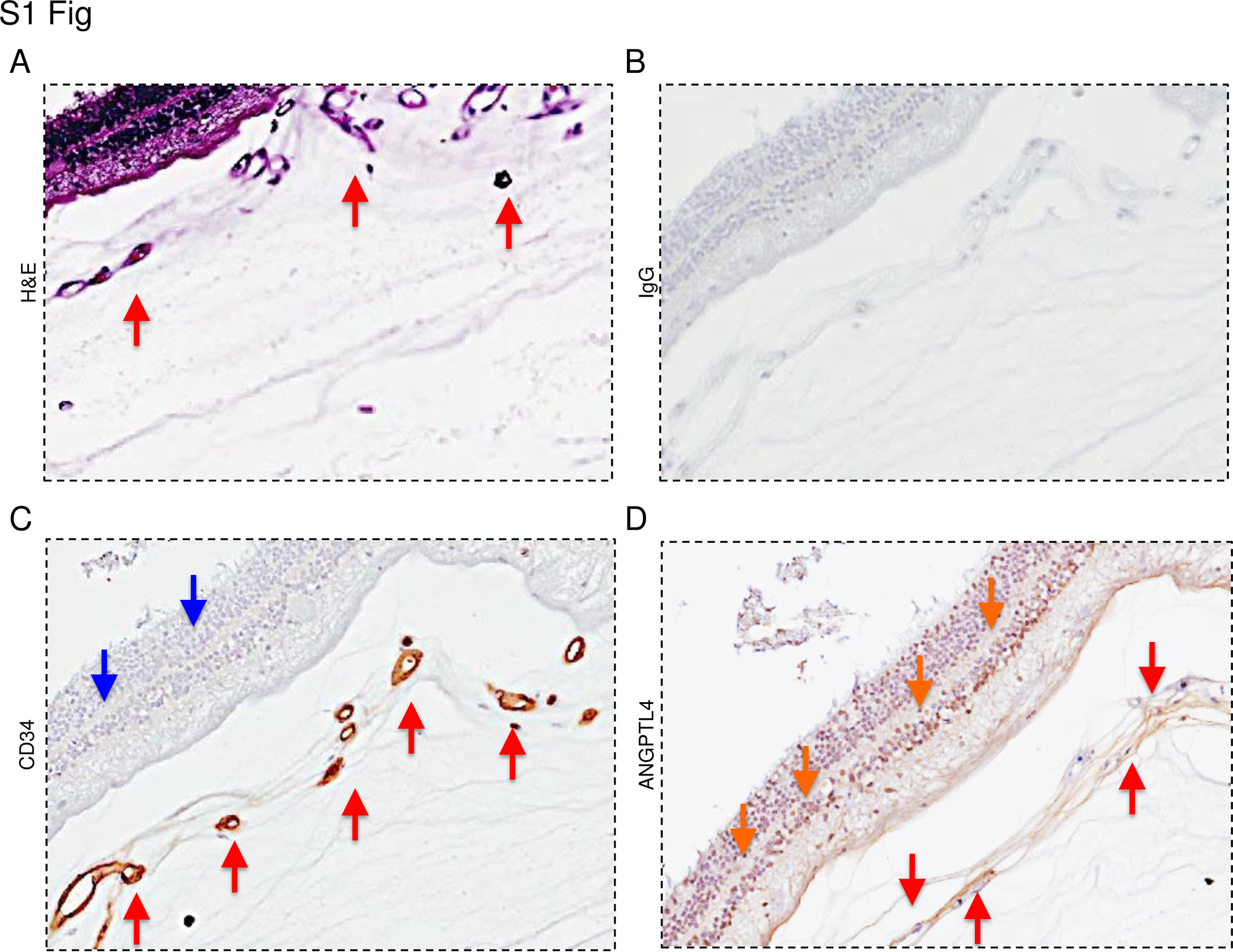

Supplement: S1 Fig — (A) H&E staining of proliferative vessels (red arrows) observed on the surface of the peripheral retina of a PSR eye. (B) Non-staining IgG (negative control). (C) CD34 staining (vascular endothelial cells) of proliferative vessels (red arrows) overlying non-perfused (i.e., no CD34 staining of retinal vessels) peripheral retina (blue arrows). (D) ANGPTL4 staining within non-perfused (i.e., no CD34 staining of retinal vessels) peripheral retina (orange arrows) and in the vascular endothelial cells and within the stroma (red arrows) of the retinal neovascular tissue overlying the peripheral non-perfused retina. (TIF) [file pone.0183320.s001.tif]

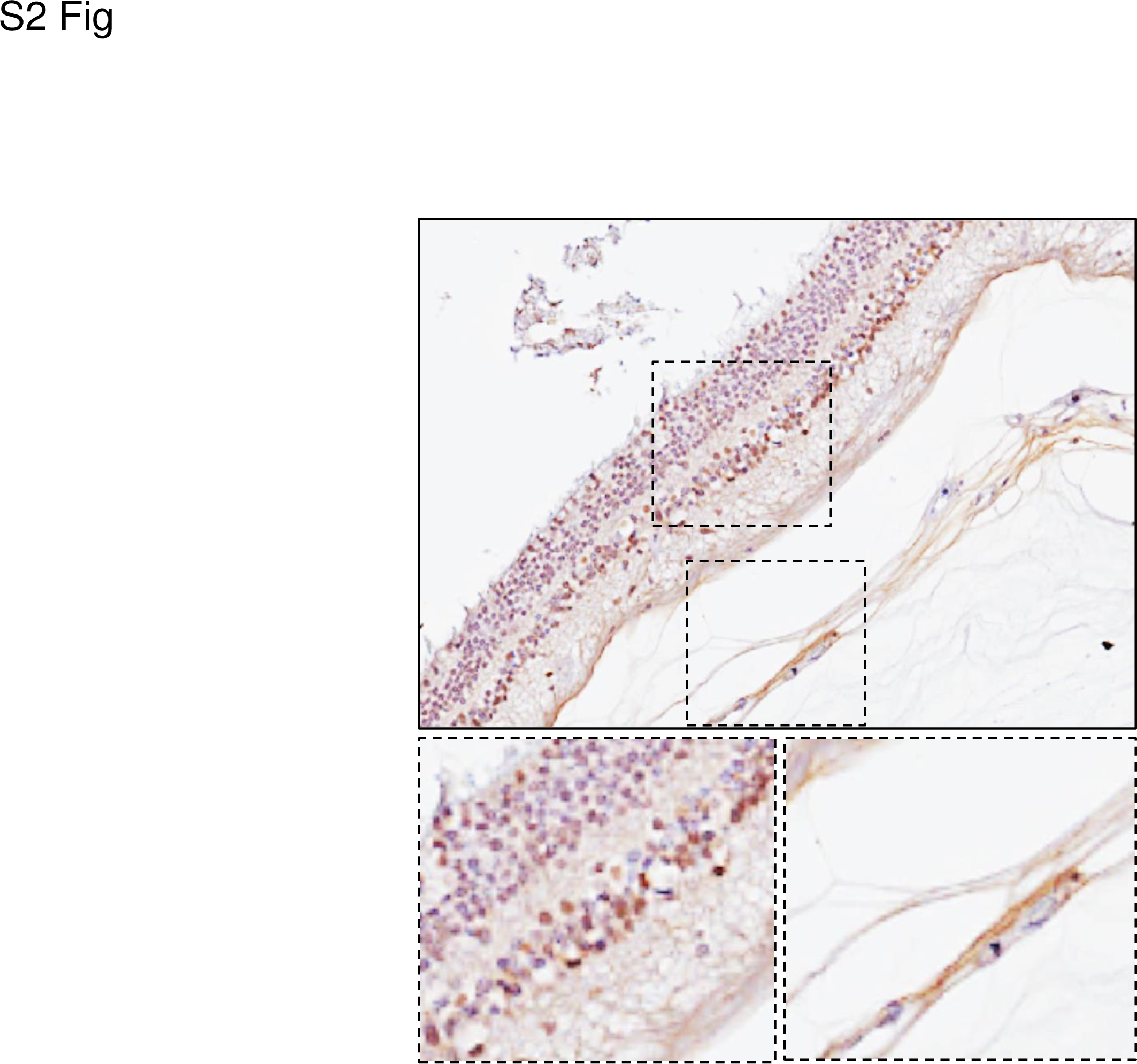

Supplement: S2 Fig — ANGPTL4 staining within inner retinal cells as well as in the vascular endothelial cells and within the stroma of the retinal neovascular tissue. (TIF) [file pone.0183320.s002.tif]
